# Mechanism of Chemical Activation of Nrf2

**DOI:** 10.1371/journal.pone.0035122

**Published:** 2012-04-25

**Authors:** Yun Li, Joseph D. Paonessa, Yuesheng Zhang

**Affiliations:** 1 Department of Cancer Prevention and Control, Roswell Park Cancer Institute, Buffalo, New York, United States of America; 2 Department of Urology, Roswell Park Cancer Institute, Buffalo, New York, United States of America; University of California Irvine, United States of America

## Abstract

NF-E2 related factor-2 (Nrf2) promotes the transcription of many cytoprotective genes and is a major drug target for prevention of cancer and other diseases. Indeed, the cancer-preventive activities of several well-known chemical agents were shown to depend on Nrf2 activation. It is well known that chemopreventive Nrf2 activators stabilize Nrf2 by blocking its ubiquitination, but previous studies have indicated that this process occurs exclusively in the cytoplasm. Kelch-like ECH-associated protein 1 (Keap1) binds to Nrf2 and orchestrates Nrf2 ubiquitination, and it has been a widely-held view that inhibition of Nrf2 ubiquitination by chemopreventive agents results from the dissociation of Nrf2 from its repressor Keap1. Here, we show that while the activation of Nrf2 by prototypical chemical activators, including 5,6-dihydrocyclopenta-1,2-dithiole-3-thione (CPDT) and sulforaphane (SF), results solely from inhibition of its ubiquitination, such inhibition occurs predominantly in the nucleus. Moreover, the Nrf2 activators promote Nrf2 association with Keap1, rather than disassociation, which appears to result from inhibition of Nrf2 phosphorylation at Ser40. Available evidence suggests the Nrf2 activators may block Nrf2 ubiquitination by altering Keap1 conformation via reaction with the thiols of specific Keap1 cysteines. We further show that while the inhibitory effects of CPDT and SF on Nrf2 ubiquitination depend entirely on Keap1, Nrf2 is also degraded by a Keap1-independent mechanism. These findings provide significant new insight about Nrf2 activation and suggest that exogenous chemical activators of Nrf2 enter the nucleus to exert most of their inhibitory impact on Nrf2 ubiquitination and degradation.

## Introduction

Nrf2 has emerged as a ubiquitous transcription factor that plays a critical role in the maintenance of cellular homeostasis. It stimulates the transcription of genes involved in many aspects of cytoprotection, most notably the Phase 2 genes, e.g., glutamate cysteine lygase (GCS) and NAD(P)H:quinone oxidoreductase-1 (NQO1). Indeed, Nrf2 knockout mice showed significantly increased susceptibility to a variety of diseases, such as cancer [1], [2], neurodegeneration [3] and inflammation [4], [5]. Nrf2 works by binding as a heterodimer with Maf or other partners to a *cis*-acting DNA regulatory element, namely the antioxidant response element (ARE), which is located in the upstream region of the target genes, stimulating gene transcription. Nrf2 is activated itself by many chemopreventive agents and is essential for some of these agents to prevent cancer and other diseases in animal models [1], [2], [6]. Consequently, pharmacological activation of Nrf2 has been widely advocated as a major strategy for prevention of cancer and other diseases [7], [8]. Interestingly, recent studies have also shown that Nrf2 may be upregulated in cancer cells and that the cytoprotective function of Nrf2 may contribute to the survival and growth of cancer cells, suggesting that it may be important to inhibit Nrf2 during cancer chemotherapy [9].

However, the mechanism by which chemopreventive agents activate Nrf2 remains less understood. While most studies have indicated that chemopreventive agents activate Nrf2 by blocking its degradation at the protein level, there are also studies suggesting that *nrf2* gene transcription may be stimulated [10], [11]. Nrf2 protein upon synthesis is rapidly degraded by the 26S proteasome in unstimulated cells (half-life of approximately 15 min) [12], [13]. Keap1, also known as the Nrf2 repressor, is crucial for the rapid turnover of Nrf2 and functions as an adaptor for Nrf2 ubiquitination at the lysine residues of the Neh2 domain by a Cul-3-dependent ubiquitin ligase complex [14], [15]. Chemical binding or oxidation of specific reactive cysteine residues of Keap1 disrupts Keap1-mediated Nrf2 ubiquitination and results in Nrf2 accumulation/activation, which in turn leads to increased transcription of ARE-regulated genes and increased cytoprotection [16]. However, there are conflicting views as to how chemical agents block Nrf2 ubiquitination. While it has been widely believed that reaction of Nrf2 activators with critical cysteine residues of Keap1 causes it to free Nrf2, thereby stabilizing Nrf2 [13], [17], [18], there are also studies suggesting that chemical modification of Keap1 cysteines is not sufficient to disrupt Nrf2 binding to Keap1 [19]–[21], and other studies report that Nrf2 phosphorylation (at Ser40) by protein kinase C or transmembrane protein kinase PERK promotes its dissociation from Keap1 [22]–[24]. Moreover, it has also been reported that chemical modification of Keap1 cysteines may trigger its own ubiquitination and degradation, freeing Nrf2 from degradation [25]. Uncertainty also exists as to where in the cell chemical activators inhibit Nrf2 degradation. The prevailing view has been that inhibition of Keap1-mediated Nrf2 ubiquitination and degradation occurs exclusively in the cytoplasm [16], [26], [27], but Nrf2 was shown to be primarily a nuclear protein [28].

**Figure 1:**
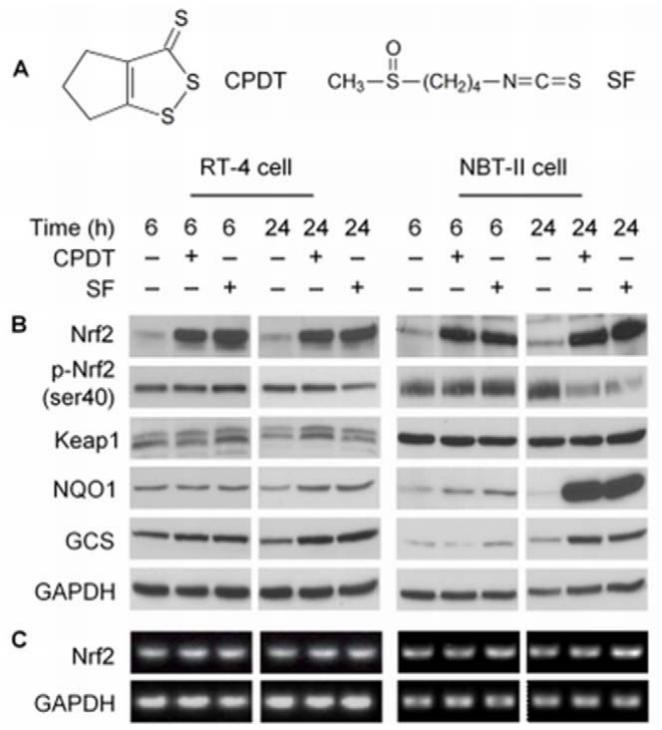
CPDT and SF stimulate Nrf2 transactivation activity by stabilizing its protein. (**A**) Chemical structure of CPDT and SF. RT-4 cells and NBT-II cells were treated with CPDT (50 µM), SF (8 µM) or vehicle (0.1% DMSO) for 6 or 24 h. (**B**) Whole cell lysates were then prepared for IB or (**C**) total RNA was isolated for RT-PCR analysis. Glyceraldehyde 3-phosphate dehydrogenase (GAPDH) was used as a control.

The present study was undertaken to further understand the mechanism of chemical activation of Nrf2. The study was carried out in multiple human and animal cell lines, utilizing CPDT and SF as prototypical Nrf2 activators. Both CPDT and SF (see **Figure 1A** for their chemical structures) are well-known chemopreventive agents and represent two major classes of chemopreventive Nrf2 activators: dithiolethiones and isothiocyanates [29]–[31]. We show that both CPDT and SF rapidly elevated Nrf2 protein, which was accompanied by increased Nrf2 transactivation activity, but did not modulate *nrf2* gene transcription and Keap1 protein expression. We further show that CPDT and SF inhibited Keap1-dependent Nrf2 ubiquitination, but Nrf2 was also degraded via a Keap1-independent pathway. Neither CPDT nor SF disassociated the Nrf2-Keap1 complex or disrupted the ubiquitin ligase complex, but both compounds inhibited Nrf2 phosphorylation, which may account for the sustained association of Nrf2 with Keap1. Moreover, we demonstrate that the machinery for Nrf2 ubiquitination and proteasomal degradation exists in both cytoplasm and nucleus but that inhibition of Nrf2 ubiquitination by CPDT and SF occurs primarily in the nucleus, rather than in the cytoplasm. These findings significantly advance our understanding about the mechanism of Nrf2 activation and also have important implication for the development of Nrf2-based chemopreventive strategies. Our data indicate for the first time that Nrf2 chemopreventive agents enter the nucleus to inhibit nuclear Nrf2 ubiquitination.

## Results

### CPDT and SF stabilize Nrf2 protein and inhibit its phosphorylation

**Figure 2:**
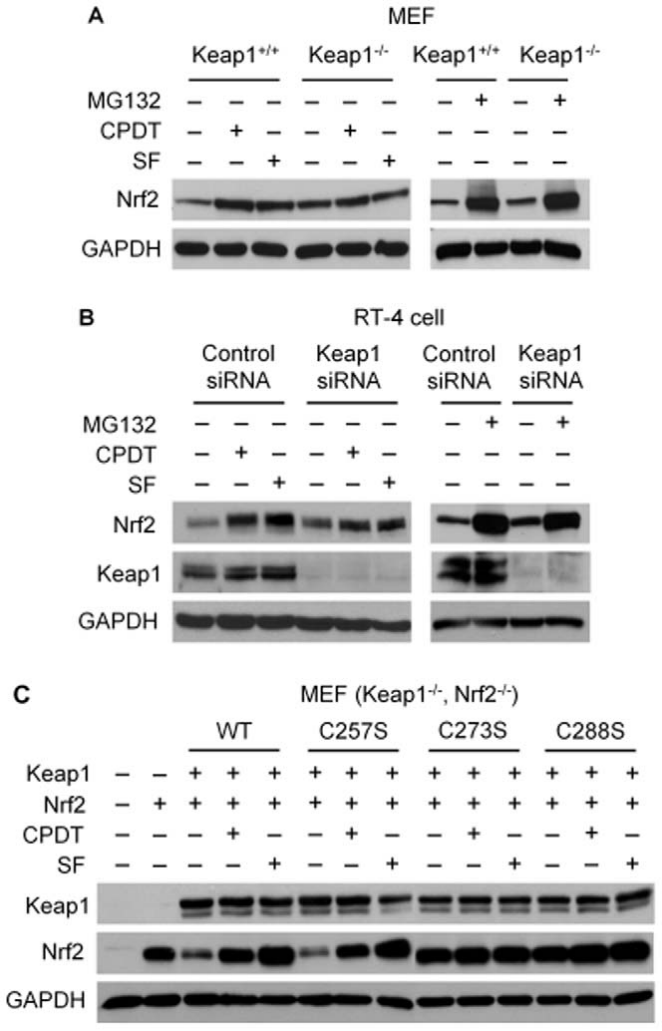
CPDT and SF block only Keap1-mediated Nrf2 degradation and require key cysteine residues of Keap1. (**A**) Murine embryonic fibroblasts (MEF), including wild type MEF (Keap1^+/+^) and MEF with Keap1 knockout (Keap1^−/−^), were treated with vehicle, MG132 (25 µM), CPDT (50 µM) or SF (8 µM) for 6 h. Cells were then harvested for IB of Nrf2 and GAPDH. (**B**) RT-4 cells were transfected with either a control siRNA or a specific Keap1-targeting siRNA for 48 h, followed by treatment with vehicle, MG132 (25 µM), CPDT (50 µM) or SF (8 µM) for 6 h. Cells were then harvested for IB of Nrf2, Keap1 and GAPDH. (**C**) MEF with knockout of both Keap1 and Nrf2 were mock transfected or transfected with expression vectors of Nrf2 (pEF/Nrf2) [42] with or without Keap1 (wild type or one of three Keap1 mutants, all cloned to pcDNA3) [32] for 48 h, followed by treatment with vehicle, CPDT (50 µM) or SF (8 µM) for 6 h. Whole cell lysates were then prepared for IB.

**Figure 3:**
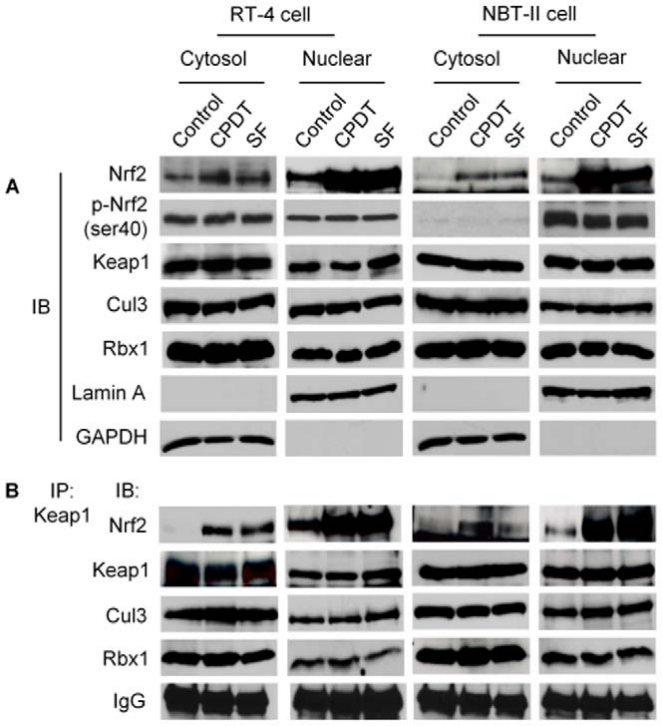
CPDT and SF block Nrf2 degradation mainly in the nucleus, but do not dissociate the Nrf2-Keap1 complex or the ubiquitination complex. RT-4 cells and NBT-II cells were treated with vehicle, CPDT (50 µM) or SF (8 µM) for 6 h, from which cytosols and nuclear extracts were prepared and were subjected to analysis by IB or IP followed by IB. The loading amount of the nuclear sample was about half of the cytoplasmic sample for both IP and IB. (**A**) IB of indicated proteins. GAPDH and lamin A were used to confirm the purity of the cytosols and nuclear extracts, respectively. (**B**) Cytosols and nuclear extracts were subjected to IP with anti-Keap1, followed by IB of the indicated proteins.

Both CPDT and SF rapidly elevated Nrf2 protein in RT-4 cells (a human bladder cancer cell line) and NBT-II cells (a rat bladder cancer cell line) (**Figure 1B**). Maximal elevation of Nrf2 appears to be reached in both cell lines after 6 h treatment with each agent. A preliminary experiment showed that both agents caused dose-dependent elevation of Nrf2; their optimal concentrations (CPDT at 50 µM and SF at 8 µM) were used in the experiments reported herein. Neither agent had any impact on Nrf2 mRNA level (Fig. 1C), indicating that *nrf2* gene transcription or its message stability was not affected by the compounds. Hence, CPDT and SF stabilized Nrf2 protein. Although a previous study suggested that Nrf2 activators might stabilize Nrf2 by increasing the ubiquitination and proteasomal degradation of Keap1 [25], we did not find that increased Nrf2 stability in response to CPDT and SF was associated with Keap1 degradation, as Keap1 remained essentially unchanged in cells treated by CPDT or SF (**Figure 1C**), Two Keap1 bands were detected in RT-4 cells, but only one in NBT-II cells. The exact reason for the doublet is not known, but such doublet was also detected in cells after transient transfection of a Keap1-expressing plasmid (**Figure 2C**) and was apparently present in other cells [26]. Previous studies have shown that Nrf2 phosphorylation at Ser40 is necessary for Nrf2 to dissociate from Keap1 and to escape Keap1-mediated ubiquitination [22]–[24]. While p-Nrf2 (p-Ser40) was readily detected in both cell lines when un-stimulated, it changed minimally at 6 h and decreased at 24 h after treatment with CPDT or SF, in contrast to the marked increase in total Nrf2 level (**Figure 1B**). Likewise, p-Nrf2 level did not change in either the cytoplasm or the nucleus, despite marked increase in Nrf2 level in both compartments in response to CPDT and SF (**Figure 3A**). Thus, both compounds are highly effective inhibitors of Nrf2 phosphorylation. This also ruled out the possibility that Nrf2 stabilization by CPDT and SF was due to phosphorylation-induced escape of Nrf2 from Keap1.

As expected, Nrf2 elevation by CPDT and SF was accompanied by significantly increased expression of GCS (heavy subunit) and NOQ1 (**Figure 1B**), both of which are Nrf2 target genes and were measured to assess Nrf2 transactivation activity. Likewise, in cells stably transfected with a Nrf2 reporter construct, both CPDT and SF significantly stimulated the reporter expression (**Figure S1**).

### Nrf2 is degraded through both Keap1-dependent and –independent pathways, but CPDT and SF only block Keap1-dependent Nrf2 degradation

As expected, Keap1 knockout in murine embryonic fibroblasts (MEF) or Keap1 knockdown by siRNA in RT-4 cells resulted in increased expression of Nrf2 (**Figure 2A** and **Figure 2B**). Treatment of MEF/Keap1^+/+^ cells or RT-4 cells (pretreated with control siRNA) with either CPDT (50 µM) or SF (8 µM) for 6 h led to significant elevation of Nrf2 protein. In contrast, the inductive effects of CPDT and SF were either absent in MEF/Keap1^−/−^ or greatly attenuated in RT-4 cells with Nrf2 knockdown (**Figure 2A** and **Figure 2B**). However, after treatment with MG132 (25 µM, 6 h), a specific proteasome inhibitor, Nrf2 was significantly elevated in both cell lines, regardless of Keap1 status (**Figure 2A** and **Figure 2B**). Similar results were obtained with epoxomicin, another specific proteasome inhibitor (result not shown). These results reveal that Nrf2 is degraded through both Keap1-dependent and Keap1-independent proteasomal pathways, but both CPDT and SF only block Keap1-mediated Nrf2 degradation.

Previous studies have identified two cysteine residues of Keap1 (C273 and C288) to be critical for Nrf2 stabilization by chemical activators [17], [32]. To confirm that Nrf2 stabilization by CPDT and SF requires these cysteines, MEF with double knockout of Keap1 and Nrf2 were transfected with a Nrf2 expression vector with or without a Keap1 expression vector for 48 h, followed by treatment with CPDT (50 µM) or SF (8 µM) for 6 h. Wild type Keap1 and three Keap1 mutants (the cysteine at 257, 273 or 288 was replaced with a serine) were tested. The C257S mutant was included as a control. Similar Keap1 expression levels were detected after transfection of each of the four Keap1 vectors (**Figure 2B**). As expected, both wild type Keap1 and the C257S mutant significantly reduced Nrf2 level, whereas both C273S and C288S mutants failed to do so. The point mutations did not affect the association of Keap1 with Nrf2 (**Figure S2**). Moreover, both CPDT and SF prevented wild type Keap1 and the C257S mutant from reducing Nrf2, but their effects on Nrf2 in cells transfected with either the C273S mutant or the C288S mutant were marginal. Given that both C273 and C288 were previously shown to be directly bound by dexamethasone 21-mesylate, a Nrf2 activator, through chemical reaction with the thiol groups [18] and that both CPDT and SF are thiol-reactive, there is little doubt that direct binding to the thiol groups of C723 and C288 of Keap1 by CPDT and SF leads to stabilization of Nrf2.

### CPDT and SF stabilize Nrf2 predominantly in the nucleus and do not disrupt Keap1-Nrf2 association

Contrary to the wide-spread belief that inhibition of Nrf2 degradation by Nrf2 activators occurs exclusively in the cytoplasm, our experiments showed that inhibition of Nrf2 degradation by both CPDT and SF took place predominantly in the nucleus, as described below. Cytoplasmic and nuclear fractions were carefully prepared from both RT-4 cells and NBT-II cells after treatment with CPDT (50 µM) or SF (8 µM) for 6 h. No cross-contamination was detected, as the nuclear marker lamin A was not detected in the cytoplasmic fraction and the cytoplasmic marker GAPDH was not detected in the nuclear fraction (**Figure 3A**). Marked Nrf2 accumulation was detected in the nucleus in both cell lines after treatment with each compound, whereas increase in cytoplasmic Nrf2 was relatively limited. It should be noted that twice as much protein was used for the cytosolic samples, compared to the nuclear samples, in the experiment shown in Fig. 3. Keap1 protein was detected in both cytoplasm and nucleus, but neither CPDT nor SF had any effect on its expression level, consistent with our result from the whole cell lysates (**Figure 1A**).

Using IP with anti-Keap1, followed by immunoblotting (IB) with anti-Keap1 or anti-Nrf2, we again found that neither CPDT nor SF had any effect on Keap1 protein level either in the cytoplasm or in the nucleus in both cell lines, but both agents significantly increased the Keap1-Nrf2 complex, mainly in the nucleus (**Figure 3B**). Comparison of the total levels of Nrf2 and Keap1 (**Figure 3A**) with their levels in the Keap1-Nrf2 complex (**Figure 3B**) suggests that most if not all of the Nrf2 molecules that were elevated by CPDT and SF were associated with Keap1. These results challenge the existing theory that Nrf2 activators stabilize Nrf2 protein by freeing it from Keap1 or by stimulating the degradation of Keap1. Increased association of Nrf2 with Keap1, induced by CPDT and SF, was not accompanied by corresponding increase in Nrf2 phosphorylation at Ser40 (**Figure 1B** and **Figure 3A**), indicating that Nrf2 phosphorylation was inhibited by these compounds in both cytoplasm and nucleus. Phosphorylation at this site was previously shown to be required for Nrf2 release from Keap1 [22]–[24].

### CPDT and SF inhibit Nrf2 ubiquitination in both cytoplasm and nucleus but do not disassociate the ubiquitin ligase complex

**Figure 4:**
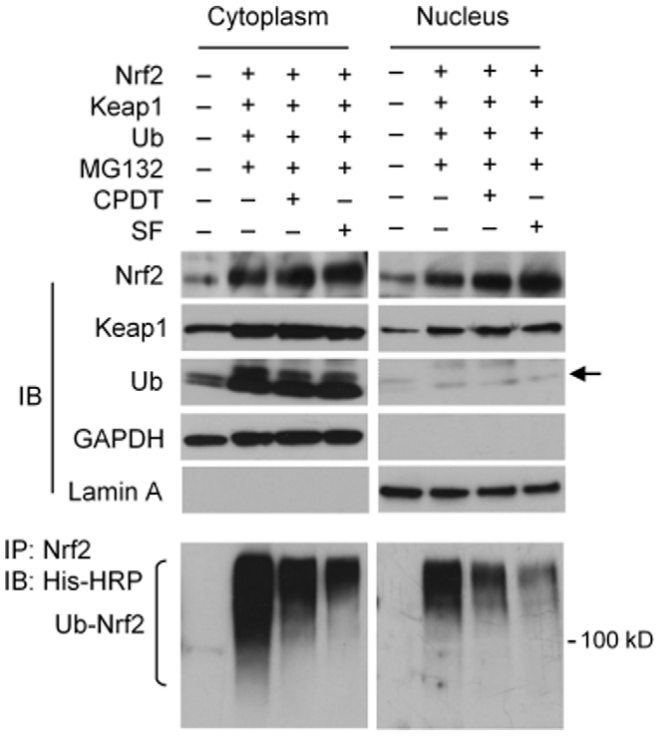
CPDT and SF block Nrf2 ubiquitination. NBT-II cells were co-transfected for 24 h with expression plasmids for Nrf2 (pEF/Nrf2), Keap1 (pcDNA1/Keap1) or ubiquitin (pMT107-His-Ub, a polyhistidine-tagged ubiquitin expression plasmid), followed by treatment for 6 h with MG132 (25 µM), MG132 (25 µM) plus CPDT (50 µM), or MG132 (25 µM) plus SF (8 µM), from which cytoplasmic and nuclear samples were prepared and analyzed by IB of various proteins. For detection of ubiquitinated Nrf2, the samples were prepared under denatured conditions and then subjected to IP with anti-Nrf2, followed by IB with an anti-His-HRP-conjugated antibody (for detection of ubiquitinated Nrf2). Equal amounts of nuclear and cytoplasmic proteins were used. The arrow points to the Ub band.

Cellular level of ubiquitinated Nrf2 was not detectable under basal conditions, with or without treatment by CPDT or SF. Thus, cells were co-transfected with three plasmids (plasmids expressing Nrf2, Keap1 and His-tagged ubiquitin) for 24 h, followed by treatment with CPDT or SF for 6 h in the presence of MG132 (to inhibit degradation of ubiquitinated Nrf2), from which both cytosolic and nuclear fractions were prepared. We focused on NBT-II cells, because ectopic expression of ubiquitin (Ub) in RT-4 cells was extremely poor. IB analysis showed that sample preparation was satisfactory, as the cytoplasmic protein GAPDH was not detected in the nuclear samples and the nuclear protein lamin A was not detected in the cytosolic samples (**Figure 4**). Treatment of NBT-II cells with the three plasmids and MG-132 significantly increased Nrf2 levels in both cytoplasmic and nuclear fractions, and CPDT and SF elevated Nrf2 level further. Keap1 levels in both cytoplasm and nucleus were increased after plasmid transfection and treatment with MG132, but neither CPDT nor SF showed any effect on Keap1. While Ub expression was not affected by CPDT and SF either, ectopic Ub expression was markedly higher in the cytoplasm than in the nucleus (**Figure 4**). Next, ubiquitinated Nrf2 was measured by IP with an anti-Nrf2 antibody and IB with a His-HRP conjugated antibody (detecting the His tag of Ub). In cells transfected with the triple plasmids and treated with MG132, ubiquitinated Nrf2 was detected in both the cytoplasm and the nucleus, but its level in the nucleus was much lower than in the cytosol, apparently due to the fact that ectopically expressed Ub existed predominantly in the cytosol (**Figure 4**). However, both CPDT and SF markedly inhibited Nrf2 ubiquitination in both the cytoplasm and the nucleus.

We next asked how CPDT and SF prevented Keap1 from promoting Nrf2 ubiquitination. It has been previously shown that Keap1 and Nrf2 form a ubiquitin ligase core complex with Cul3 and Rbx1 in the cytoplasm [15]. However, all four proteins were detected in the cytoplasm and nucleus of both RT-4 cells and NBT-II cells, and neither CPDT nor SF had any effect on the expression of Keap1, Cul3 and Rbx1 in both compartments (**Figure 3A**). Moreover, in experiments involving IP with anti-Keap1, followed by IB with respective antibodies, neither CPDT nor SF disrupted the association of Keap1 with Cul3 and Rbx1 in either cytoplasm or nucleus, while Nrf2 level in the complex increased significantly, especially in the nucleus (**Figure 3B**). These results show that a Nrf2 ubiquitination-proteasome degradation system is present in both cytoplasm and nucleus, and that inhibition of Nrf2 ubiquitination by CPDT and SF does not result from the disassociation of the ubiquitination core complex.

## Discussion

In the present study, CPDT and SF were used as probes to understand the mechanism of chemical activation of Nrf2. CPDT and SF belong to two well-known classes of Nrf2 activators - dithiolethione and isothiocyanate, many of which are being investigated for prevention of cancer and other diseases. The chemopreventive activity of SF, which occurs in broccoli and other plants is particularly promising [30]. We show that both CPDT and SF markedly elevate Nrf2 protein level and stimulate Nrf2 transactivation activity in human, mouse and rat cell lines (**Figures 1** **,** **2** **,** **3**). Similar results were previously shown in other cell lines [33], [34]. Thus, the effects of CPDT and SF on Nrf2 are neither cell line-specific nor species-specific. Neither agent showed any effect on *nrf2* gene transcription and Keap1 protein expression. Thus, the activities of these agents also fit with the widely accepted view that chemopreventive agents generally activate Nrf2 by blocking its protein degradation. Based on these considerations, CPDT and SF may be viewed as prototypical Nrf2 activators. It should be noted, however, that in all of our experiments, the Nrf2 band was detected at ∼100 kD, although the expected molecular weight of Nrf2 is ∼68 kD. The exact reason is not known, but this phenomenon has been previously reported and may be related to a Nrf2-actin complex [35], [36].

Keap1 promotes proteasomal degradation of Nrf2 by orchestrating Nrf2 ubiquitination. As expected, we found that Keap1 knockout or knockdown caused significant increase in Nrf2 level, whereas forced expression of Keap1 reduced Nrf2 expression (**Figure 2**). Both CPDT and SF significantly elevated Nrf2 levels in Keap1-present cells, but such effect was completely lost in Keap1-knockout cells or greatly attenuated in cells whose Keap1 was knocked down by siRNA (**Figure 2**). Hence, the Nrf2-elevating ability of CPDT and SF depends on Keap1. This information has a practical implication in the design of chemopreventive strategies. Hayes and coworkers previously suggested that Keap1 knockdown with RNAi might be a potentially useful strategy for cancer chemoprevention [37]. Clearly, such a strategy cannot be combined with CPDT, SF or similar agents, since the ability of the latter to activate Nrf2 depends on Keap1. However, we have shown in this study that Nrf2 protein is degraded through both Keap1-dependent and Keap1-independent proteasomal pathways. At the present time, little is known about Keap1-independent proteasomal degradation of Nrf2.

Both CPDT and SF inhibited Nrf2 ubiquitination in both cytoplasm and nucleus (**Figure 4**). While Keap1 associates with Cul3 and Rbx1 to form a functional E3 ubiquitin ligase core complex, which ubiquinates Keap1-bound Nrf2 and targets it for degradation by 26S proteasome [14], [20], neither CPDT nor SF dissociates the ubiquitination core complex or alter the expression of each protein (**Figure 3**). Moreover, our data clearly show that the Nrf2-ubiquinating complex exists in both cytoplasm and nucleus and that inhibition of Nrf2 ubiquitination by CPDT and SF occurs primarily in the nucleus (**Figure 3** and **Figure 4**). It is of note that the 26S proteasome has been shown to exist in both cytoplasm and nucleus [38]. In contrast, a previous report indicated that Nrf2 was ubiquitinated and degraded only in the cytoplasm [26]. The reason for such a discrepancy is not entirely clear, but may be related to experimental conditions. We have found that forced overexpression of Keap1, Nrf2 and Ub in cultured cells, which was used in the previous study mentioned above, could generate misleading results, as ectopically expressed Keap1 and Nrf2 existed largely in the cytoplasm in MEF (**Figure S2**) and ectopically expressed Ub existed largely in the cytoplasm in NBT-II cells (**Figure 4**). We have also shown that inhibition of Nrf2 ubiqutination by CPDT or SF does not result from Nrf2 release from Keap1; rather, both agents prevent Nrf2 from leaving Keap1 (**Figure 3**). This also directly contrasts with the widely-accepted model that chemical activation of Nrf2 results from dissociation of Nrf2 from Keap1, thereby allowing Nrf2 to escape Keap1-mediated proteasomal degradation. It is particularly surprising that the Keap1-Nrf2 dissociation model was based in part on studies involving SF [18]. The reason for the discrepancy is not entirely known, but a close examination of the previous studies that led to the Keap1-Nrf2 dissociation model revealed that key experiments were performed in cell-free systems [18], [26]. In addition, our present study also appears to have uncovered the potential mechanism by which CPDT and SF prevent Nrf2 release from Keap1. Both CPDT and SF strongly inhibited Nrf2 phosphorylation at Ser40 (**Figure 1B** and **Figure 3A**). It was previously shown that phosphorylation at this site is required for Nrf2 release from Keap1 [22]–[24]. However, it is not yet known if CPDT and SF inhibit the phosphorylation of and/or stimulate the dephosphorylation of Nrf2. A putative nucleocytoplasmic shuttling system of Keap1/Nrf2 has been previously reported [26], but neither CPDT nor SF had any effect on Keap1 level in either the cytoplasm or the nucleus of RT4 cells and NBT-II cells (**Figure 3**), implying that this shuttle system was not involved in CPDT- and SF-induced increase in nuclear Nrf2. A previous study also found that compounds such as diethyl maleate and butylated hydroxyanisole promoted nuclear accumulation of Nrf2 without altering the subcellular localization of Keap1 [27].

**Figure 5:**
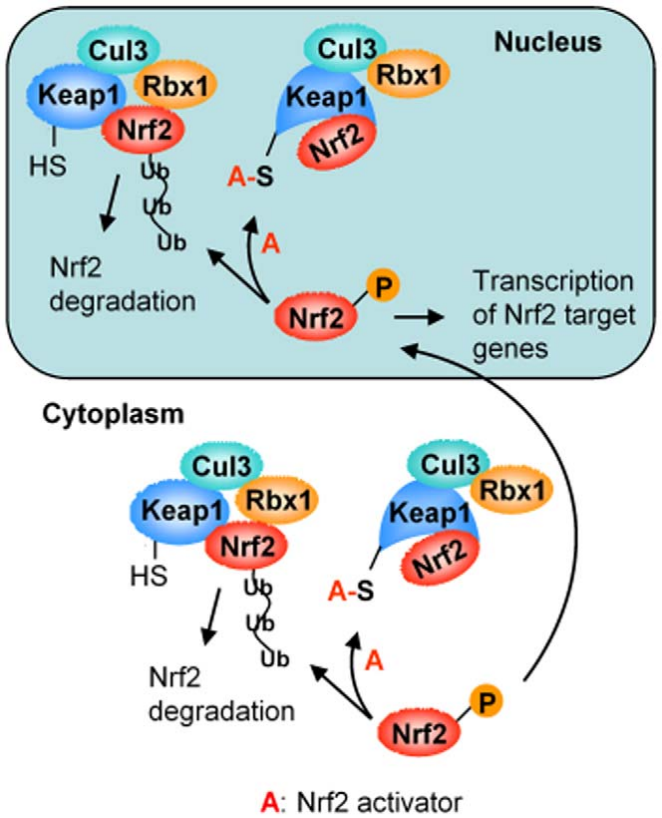
The paradigm of chemical activation of Nrf2. The Keap1-mediated Nrf2 ubiquitination and proteasomal degradation exist in both cytoplasm and nucleus. Nrf2 activators block Nrf2 ubiquitination by causing conformational change of Keap1 through reaction with its critical cysteine residues (C273 and C288), and this process occurs primarily in the nucleus. Keap1 is shown as a monomer in this model, but a previous study suggests that Nrf2 may be associated with Keap1 homodimer [43].

In summary, both CPDT and SF inhibit Keap1-mediated Nrf2 ubiquitination, and such inhibition occurs mainly in the nucleus (**Figure 5**). Neither agent disrupts the association of the Nrf2-ubiquitinating core complex, nor do they free Nrf2 from Keap1 or affect the potential nucleocytoplasmic shuttling of Keap1/Nrf2. Cys273 and Cys288 of Keap1 are essential for the Nrf2-stabilizing activity of CPDT and SF; these cysteines likely undergo conjugation reaction through their thiol groups with CPDT and SF or their metabolites. We propose that such reactions cause conformational change of Keap1 and renders Keap1-bound Nrf2 unreachable by the ubiquitin ligase (**Figure 5**). Indeed, a previous study suggests that C273 and C288 of Keap1 function as ligands in zinc coordination [39]. It is conceivable that modification of these cysteines by CPDT and SF as well as other Nrf2 activators disrupts the zinc coordination and thus alters Keap1 conformation.

## Materials and Methods

### Antibodies and reagents

The antibodies were purchased from Santa Cruz Biotechnology (Santa Cruz, CA), including anti-Nrf2 (sc-722, sc-13032), anti-GCS (sc-22755, detecting GCS heavy subunit), anti-Keap1 (sc-15246), anti-lamin A (sc-20680), anti-ubiquitin (sc-8017) and anti-His-tag (His-probe H3), and from Millipore (Billerica, MA), including anti-Rbx1 (AB3737) and anti-GAPDH (MAB374). Other antibodies including anti-NQO1 (3187), anti-p-Nrf2 (2073-1; specifically recognizing phosphorylation at serine 40) and anti-Cul3 (611848) were purchased from Cell Signaling (Beverly, MA), Epitomics (Burlingame, CA), BD Biosciences (San Jose, CA), respectively. An anti-His-HRP-conjugated antibody was purchased from Qiagen (Valencia, CA). CPDT was provided by Dr. Rex Munday [32]. SF and epoxomicin were purchased from LKT Laboratories (St. Paul, MN), and MG132 was purchased from EMD (Gibbstown, NJ).

### Cell culture, chemical treatment, gene knockdown and gene overexpression

NBT-II cells [33], RT-4 cells [40], MEFs (wild type MEF, MEF/Keap1^−/−^, MEF/Keap1^−/−^/Nrf2^−/−^) [17] were cultured in MEM, Iwakata and Grace's modified McCoy's 5A, and Iscove's modified DMEM, respectively, at 37°C and 5% CO_2_. All media were supplemented with 10% FBS. For treatment with the test agents, cells were seeded in 10-cm dishes (1–2×10^6^ cells/10 ml medium/dish) or 6-well plates (0.25×10^6^ cells/2 ml medium/well) overnight and then treated with a test agent for a desired time before harvest by trypsin treatment and low-speed centrifugation at 4°C. All agents were dissolved in DMSO, and the final DMSO concentration in the media was <0.25% (v/v). To knock down Keap1, cells were transfected with Keap1 stealth RNAi (HSS190639) or a universal control stealth RNAi (12935300) from Invitrogen (Carlsbad, CA) with lipofectamine 2000 for 48 h, following the manufacturer's protocol. For gene overexpression, cells were transfected with a desired plasmid (2 µg DNA/well for MEF or 6 µg DNA/dish for NBT-II cells) for 24–48 h, according to the manufacturer's recommended protocol for plasmid transfection with lipofectamine 2000.

### Subcelluar fractionation, immunoprecipitation and immunoblotting

Cytoplasmic and nuclear fractions were prepared using NE-PER Nuclear and Cytoplasmic Extraction Reagents Kit (Thermo Scientific, Waltham, MA), following the manufacturer's instruction. For immunoprecipitation (IP), cytosols and nuclear extracts were incubated with a desired IP antibody, and the immune complexes were pulled down by incubation with protein A or G sepharose 4 Fast Flow Beads (GE Healthcare, Piscataway, NY) and centrifugation. When measuring ubiquitinated Nrf2, the cytosols and nuclear extracts were denatured first to disrupt potential association of Nrf2 with other proteins before IP. These and other samples were electrophoresed on SDS-PAGE and transferred to polyvinylidene difluoride membranes. The membranes were blocked in TBS-0.05% Tween 20 with 5% non-fat milk, incubated with primary antibodies, and after extensive wash, incubated with a peroxidase-conjugated secondary antibody. Protein bands were visualized using either the Amersham ECL Plus System (GE Healthcare) or the SuperSignal West Pico Chemiluminescence Detection System (Thermo Scientific).

### RT-PCR

Total RNA was isolated using the Qiagen RNeasy Mini Kit and Qiagen Qiashredder (Qiagen, Germantown, MD), from which cDNA was synthesized using random primers and SuperScript II RT (Invitrogen). PCR primers were as follows: Rat Nrf2, 5′-agt cgc ttg ccc tgg ata ttc-3′ and 5′- gcc gga gtc aga gtc att gaa-3′; human Nrf2, 5′-atg gat ttg att gac ata ctt t-3′ and 5′- act gag cct gat tag tag caa t-3′; GAPDH, 5′- gac cac agt cca tgc cat ca -3′ and 5′ – tcc acc acc ctg ttg ctg ta- 3′. PCR was carried out in a 50 µl volume and run for 30 cycles in an Eppendorf Mastercycler Gradient Thermal Cycler (Eppendorf, Hamburg, Germany). The amplified products were electrophoresed on agarose gel and stained with ethidium bromide.

## Supporting Information

Figure S1 **Stimulation of Nrf2 transactivation actvitity by CPDT and SF.** HepG2 cells were stably transfected with either a Nrf2 reporter construct (ARE-TK-GFP), where the cDNA coding the green fluorescence protein (GFP) was cloned in tandem behind the Nrf2-binding element antioxidant response element (ARE) and the thymidine kinase promoter (TK), or a control vector (TK-GFP) as previously described [41]. These cells were cultured in DMEM with 10% FBS and treated with vehicle (DMSO), CPDT (50 µM) or SF (8 µM) for 24 h. Whole cell lysates were then prepared to measure the relative GFP level using a fluorescence spectrometer as previously described [37]. Each value is a mean ± SD (n = 3). Two sided t-test was used for data analysis.
(TIF)

Figure S2 **The effects of CPDT and SF on Keap1-mediated Nrf2 degradation.** Murine embryonic fibroblasts (MEF) were cultured in Iscove's modified DMEM. MEF with knockout of both Keap1 and Nrf2 were co-transfected with expression vectors of Nrf2 and one of the two Keap1 mutants (C257S and C273S) for 48 h, followed by treatment with vehicle, CPDT (50 µM) or SF (8 µM) for 6 h. Both cytosolic fractions and nuclear fractions were prepared, using the NE-PER Nuclear and Cytoplasmic Extraction Reagents Kit (Thermo Scientific, Waltham, MA). Cross-contamination was ruled out by IB of β-tubulin (cytoplasmic marker) and lamin A (nuclear marker). Both fractions were then subjected to IP by anti-Keap1, followed by IB with anti-Nrf2 and anti-Keap1.
(TIF)
