# Supplementary figures and images for: Mechanism of Chemical Activation of Nrf2
Source: PLoS One. 2012 Apr 25;7(4):e35122. doi: 10.1371/journal.pone.0035122 (PMC3338841; doi:10.1371/journal.pone.0035122)

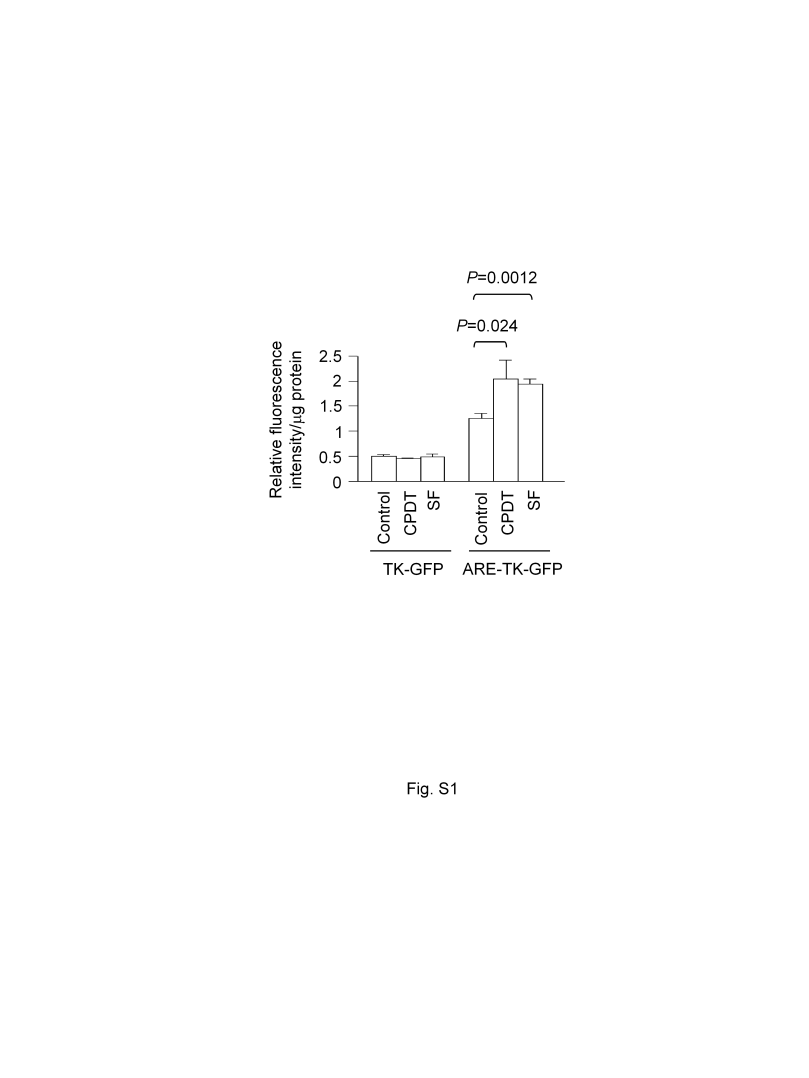

Supplement: Figure S1 — Stimulation of Nrf2 transactivation actvitity by CPDT and SF. HepG2 cells were stably transfected with either a Nrf2 reporter construct (ARE-TK-GFP), where the cDNA coding the green fluorescence protein (GFP) was cloned in tandem behind the Nrf2-binding element antioxidant response element (ARE) and the thymidine kinase promoter (TK), or a control vector (TK-GFP) as previously described [41]. These cells were cultured in DMEM with 10% FBS and treated with vehicle (DMSO), CPDT (50 µM) or SF (8 µM) for 24 h. Whole cell lysates were then prepared to measure the relative GFP level using a fluorescence spectrometer as previously described [37]. Each value is a mean ± SD (n = 3). Two sided t-test was used for data analysis. (TIF) [file pone.0035122.s001.tif]

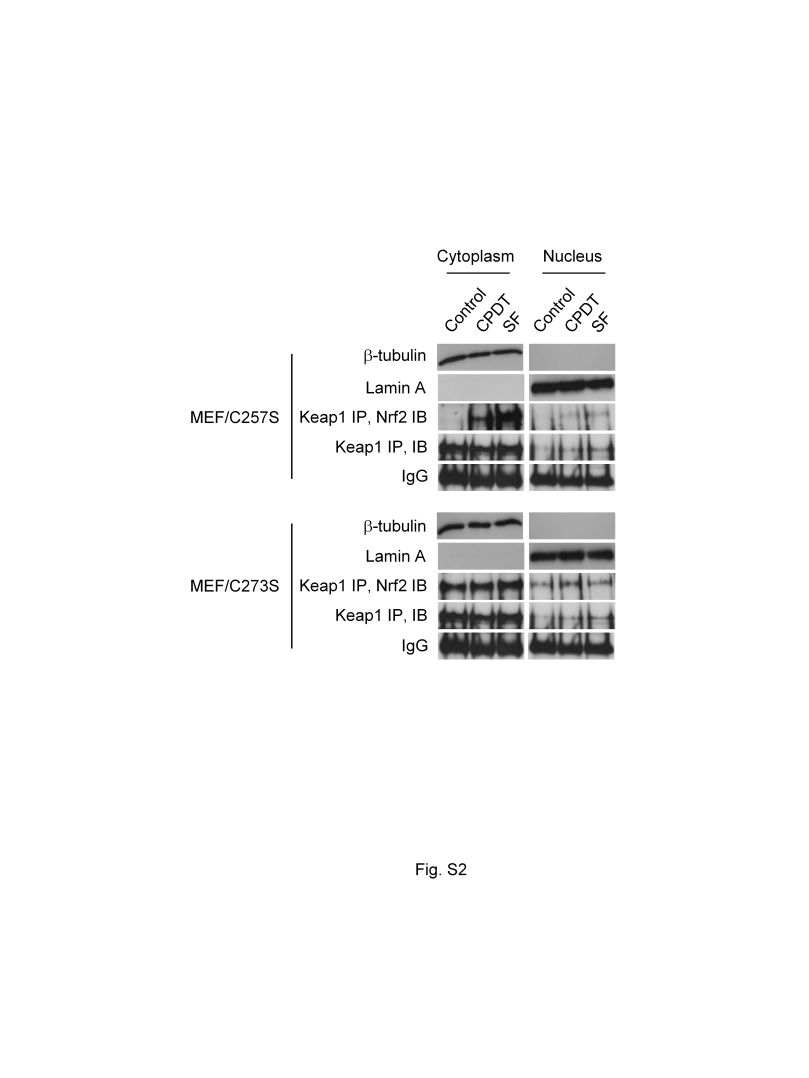

Supplement: Figure S2 — The effects of CPDT and SF on Keap1-mediated Nrf2 degradation. Murine embryonic fibroblasts (MEF) were cultured in Iscove's modified DMEM. MEF with knockout of both Keap1 and Nrf2 were co-transfected with expression vectors of Nrf2 and one of the two Keap1 mutants (C257S and C273S) for 48 h, followed by treatment with vehicle, CPDT (50 µM) or SF (8 µM) for 6 h. Both cytosolic fractions and nuclear fractions were prepared, using the NE-PER Nuclear and Cytoplasmic Extraction Reagents Kit (Thermo Scientific, Waltham, MA). Cross-contamination was ruled out by IB of β-tubulin (cytoplasmic marker) and lamin A (nuclear marker). Both fractions were then subjected to IP by anti-Keap1, followed by IB with anti-Nrf2 and anti-Keap1. (TIF) [file pone.0035122.s002.tif]
